# Temporal changes in culturable fungal community composition with their plastic degradation capacities in the marine plastisphere through mesocosm experiments

**DOI:** 10.71150/jm.2605011

**Published:** 2026-08-31

**Authors:** Sumin Jo, Ji Seon Kim, Wonjun Lee, Chang Wan Seo, Young Woon Lim

**Affiliations:** 1 School of Biological Sciences and Institute of Biodiversity, Seoul National University, Seoul 08826, Republic of Korea; 2 Global Institute for Advanced Nanoscience & Technology, Changwon National University, Changwon 51140, Republic of Korea

**Keywords:** culturable fungi, marine plastisphere, fungal community, plastic degradation, mesocosm, biofilm succession

## Abstract

Plastic waste in marine environments provides novel habitats for diverse organisms, forming distinct microbial ecosystems known as the ‘plastisphere’. Although bacteria of the plastisphere have been widely studied, the role of fungi in plastisphere formation and plastic degradation remains largely unexplored. Thus, we investigated temporal changes in culturable fungal community composition on three common plastic types—high-density polyethylene, low-density polyethylene, and polypropylene—across the early (seven days) and mature (30 days) plastisphere developmental stages through a marine mesocosm experiment. In total, 436 fungal strains were isolated and identified as belonging to 179 taxa, with *Penicillium*, *Cladosporium*, *Trichoderma*, *Aspergillus*, and *Fusarium* as the dominant genera. Temporal shifts in the species richness of the dominant genera were observed: *Cladosporium* showed higher species richness at the early stage, whereas that of *Trichoderma* increased at the mature stage. Plastic degradation assays revealed that 54.6% of the strains exhibited measurable degradation capacity, with patterns varying by plastic type rather than fungal developmental stage. Scanning electron microscope observations revealed surface damage patterns including cracks, pitting, and erosion on the plastic surfaces. These findings provide novel insights into the composition and functional heterogeneity of culturable fungal communities in the marine plastisphere and suggest that plastisphere fungi play diverse ecological roles beyond direct plastic degradation.

## Introduction

Plastic waste accumulation extensively impacts marine ecosystems (Yu et al., 2023), as 75–199 million tons of plastic waste are estimated to flow into the marine environment (UNEP, 2021). High-density polyethylene (HDPE), low-density polyethylene (LDPE), and polypropylene (PP) account for the highest global plastic production volumes (Geyer et al., 2017). Plastic wastes provide a novel habitat for a variety of organisms, which is referred to as the ‘plastisphere.’ This habitat is distinct from those of the surrounding marine microbial communities and provides a unique ecological niche through interactions with the surrounding seawater conditions (Amaral-Zettler et al., 2020; Jacquin et al., 2019; Li et al., 2024; Zettler et al., 2013).

When plastics are exposed to the marine environment, a conditioning film rapidly forms on their surfaces (Rummel et al., 2021), enabling pioneer microbes, such as bacteria and diatoms, to develop an extracellular polymeric substance (EPS)-based plastisphere, followed by the adhesion of secondary colonizers, such as ciliates, dinoflagellates, and fungi (Dobretsov et al., 2013; Qian et al., 2022). As succession progresses, sessile marine organisms such as macroalgae, barnacles, and tube worms also settle, leading to the maturation of the plastisphere (Dobretsov et al., 2013). Importantly, early microbial communities do not only initiate plastisphere formation through EPS production but may also modulate plastic surface properties and resource availability, thereby influencing both degradation processes and colonization toward the development of a mature plastisphere. Although bacterial succession on marine plastics has been studied alongside biofilm development and functional activity profiles, including increases in extracellular enzymatic activity during prolonged immersion (Odobel et al., 2021), the roles and dynamics of other microbial groups are yet to be systematically characterized.

Fungi are efficient decomposers of recalcitrant substrates (e.g., lignin and cellulose), suggesting their potential for degrading marine plastic waste. Previous research on fungi associated with the marine plastisphere has primarily focused on identifying a few strains with high plastic degradation capacities (Berger et al., 2026; Vaksmaa et al., 2024) or characterizing their plastic degradation mechanisms (Gao et al., 2022; Zhang et al., 2022). However, studies that simultaneously track temporal shifts in the compositions of marine plastisphere fungal communities and the corresponding changes in plastic degradation capacity remain limited.

Plastisphere succession has been reported to enrich degradation-related taxa and functions over time (Wang et al., 2022), and plastic-utilizing fungal isolates have more frequently been recovered after longer immersion periods (Philippe et al., 2024). Based on these observations, we hypothesized that fungal strains with strong degradation capabilities would appear more frequently during the mature stage of plastisphere development. We aimed to investigate the temporal changes in fungal community composition in marine plastispheres by comparing the culturable fungal diversity on plastic sheets submerged for seven days (early stage) and 30 days (mature stage) using a mesocosm experiment. The plastic degradation capacity of each fungal strain was evaluated through weight-loss measurements and scanning electron microscope (SEM) observations to directly assess plastic surface damage. By integrating these results, this study provides insights—including the discovery of fungal resources with the potential for bioremediation—into fungal contributions to the formation and maintenance of the marine plastisphere.

## Materials and Methods

### Mesocosm design and sample collection

Mesocosm experiments were conducted from July to August 2024 in a sea cage at the South Sea Research Institute of the Korea Institute of Ocean Science and Technology (KIOST) (34°59’33.5” N, 128°40’27.0” E). The detailed layout of the mesocosm structure is shown in Fig. 1A. Briefly, each mesocosm structure consisted of stainless-steel tea balls (prepared for each plastic type) containing four plastic sheet pieces internally connected by sterilized fishing lines, which were connected to cylindrical pots. To minimize wave disturbance, each mesocosm structure was submerged at approximately 1 m below the sea surface using buoys.

For the preparation of the mesocosm structures, three types of plastic sheets of 1 mm thickness were purchased: HDPE (Goodfellow, catalogue no. ET32-SH-000100), LDPE (ET31-SH-000110), and PP (PP30-SH-000100). Plastic sheets were cut into 8 × 2 cm pieces and surface-sterilized by sequential washing with 70% (v/v) ethanol (Samchun Chemicals, Republic of Korea) and distilled water, followed by overnight exposure to UV light (253.7 nm). Stainless-steel tea balls and fishing lines were sterilized by autoclaving.

One unit of each mesocosm was collected at seven and 30 days after deployment (one unit per plastisphere developmental stage). The plastic sheets in each tea ball were rinsed three times on site with sterilized distilled water (DW) and subjected to different pretreatments, depending on the subsequent experimental objectives. For the fungal isolation experiments, each plastic sheet (nine replicates/plastic type) was immersed in a modified fungal inoculation solution to maintain fungal cell viability and facilitate cell detachment. The solution consisted of 30% (v/v) glycerol (Junsei Chemical, Japan) and 0.05% (w/v) agar (BD Difco^TM^, Detroit, USA) prepared in sterilized seawater (SSW; instead of distilled water) supplemented with 0.05% (v/v) Tween 80 (Sigma-Aldrich, USA) as a surfactant. For the microscopic observation of plastisphere colonization and plastic surface alterations, each plastic sheet (two replicates/plastic type) was immersed in SSW. All pretreated samples were transported to the laboratory at 4℃.

### Fungal isolation

The sampled plastic sheets were immersed in the modified fungal inoculation solutions and vortexed at maximum speed for 10 min to detach biofilms from the plastic sheet surfaces. For fungal isolation, the resulting homogenates were filtered through 0.2-μm polycarbonate track-etched membrane filters (GVS Filter Technology, USA) to collect fungal cells. The membrane filters were then placed onto three different isolation media: marine agar (MA; BD Difco^TM^, USA), minimal medium (MM; 2.0 g NaNO_3_, 0.7 g KH_2_PO_4_, 0.3 g K_2_HPO_4_, 0.5 g KCl, 0.5 g MgSO_4_·7H_2_O, 0.1 g FeSO_4_·7H_2_O, and 30 g agar/L of SSW) (Mathur and Prasad, 2012), and yeast extract peptone dextrose agar (YPDA; BD Difco^TM^, USA). For each plastic type, three replicate filters were used, with one filter placed on each medium type and incubated at 25℃ for two weeks. To obtain pure cultures, single colonies were subcultured on fresh potato dextrose agar (PDA; BD Difco^TM^, USA) supplemented with SSW. Each pure fungal culture was preserved as 20% glycerol stocks supplemented with SSW and stored at –20℃ in the Seoul National University Fungal Collection.

### Molecular identification

Genomic DNA was extracted from the fresh mycelia of each PDA fungal culture using an AccuPrep Genomic DNA Extraction kit (Bioneer, Republic of Korea), according to the manufacturer’s instructions. Polymerase chain reaction (PCR) amplifications were performed in a C1000 Thermal Cycler (Bio-Rad Laboratories, USA) using AccuPower^®^ PCR Premix (Bioneer, Republic of Korea). The internal transcribed spacer (ITS) region sequences were amplified using fungal-specific primers (PCR conditions are summarized in Table S1). PCR amplicons were verified by electrophoresis on 1% agarose gel and purified using an ExoSAP-IT Express PCR Product Cleanup kit (Thermo Fisher Scientific, USA). Sequencing was performed by Macrogen (Republic of Korea) on an ABI PRISM 3700 Genetic Analyzer (Life Technologies, USA) using the same primer sets used for PCR.

The sequences obtained were proofread, trimmed, and manually edited using Geneious Prime 2024.7.12 (https://www.geneious.com). Forward and reverse reads were assembled using the de novo assembly function in Geneious Prime to obtain consensus sequences. Preliminary identification of higher taxonomic ranks (genus level or above) was performed using NCBI BLAST searches of the ITS sequences. For taxa that were not reliably identifiable to the species level based on the ITS sequence alone, additional genetic markers were amplified and sequenced under the conditions specified in Table S1. The newly generated sequences were deposited in the GenBank database of the NCBI (accession numbers are listed in Table S2).

Reference sequences were retrieved from GenBank based on a literature search and combined with our newly obtained sequences for phylogenetic analysis. A phylogenetic tree for each genus was inferred using either (1) FunVIP v.0.4.1 with “accurate” mode and 1,000 bootstrap replicates (Seo et al., 2025), or (2) RAxML implemented in Geneious Prime with 1,000 bootstrap replicates. The final species-level identification of each strain was based on the resulting phylogeny.

### Assessment of plastic degradation capacity by weight-loss measurements

The plastic-degrading capacity of each fungal isolate was evaluated individually using an in vitro degradation assay. To assess the plastic degradation capacities of fungi, 6 mm diameter fungal mycelial discs cut from one-week-old PDA fungal cultures supplemented with SSW were inoculated at the center of MM plates. Plastic sheets (HDPE, LDPE, and PP) were punched into discs of 6 mm in diameter and surface-sterilized using the same methods as those used for the mesocosm plastic sheets. Sterilized plastic discs were placed approximately 15 mm from each fungal inoculum and served as the sole carbon source. Each strain was tested only against the plastic type from which it was originally isolated. After 60 days of incubation at 25℃, plastic discs were washed to remove adherent mycelia by sequential washing with 30% (w/v) sodium dodecyl sulfate (Sigma-Aldrich, USA), 70% (v/v) ethanol (Samchun Chemicals, Republic of Korea), and DW (Sathiyabama et al., 2024). Subsequently, the discs were oven-dried at 40°C for two to three days, and each disc was weighed using an electronic balance (EPG213C, Ohaus Co., USA). The control samples consisted of plastic discs without fungal inoculation that underwent identical incubation, washing, and measurement procedures.

Plastic degradation capacity was assessed by weight-loss percentage, calculated as


$$\mathrm{Wight}\;\mathrm{loss}\;\mathrm{percentage}\;\left(\%\right)\;=\;\frac{\mathrm{Initial}\;\mathrm{weight}-\mathrm{Final}\;\mathrm{weight}}{\mathrm{Initial}\;\mathrm{weight}}\times 100,$$

with all values presented as Mean ± Standard Deviation relative to the controls. In cases where experimental discs showed weight gain relative to controls, the weight loss percentage (%) was adjusted to 0.

### Scanning electron microscopic analysis

To visually examine plastic degradation patterns by fungi, plastic samples were analyzed using a field-emission SEM (FE-SEM) at the National Instrumentation Center for Environmental Management (NICEM), Seoul National University (Republic of Korea). Two types of plastic samples were analyzed: (1) plastic sheets from mesocosm experiments (two sheets/plastic type at each stage) and (2) plastic discs from degradation assays inoculated with the fungal strain showing the highest degradation capacity. For the latter samples (plastic discs), adherent mycelia were removed prior to fixation by sequential washing with 30% (w/v) sodium dodecyl sulfate (SDS; Sigma-Aldrich, USA), 70% ethanol, and DW (with vortexing for 20 min after each step). All samples were subsequently fixed in 3% (v/v) glutaraldehyde (Thermo Fisher Scientific, USA) for 24–48 h and rinsed three times in DW at room temperature (10 min/wash). Fixed samples were dehydrated using a graded ethanol series (30%, 50%, 70%, 80%, and 90%; 10 min in each solution, with two final exchanges at 100%), followed by one treatment with a 1:1 (v/v) mixture of ethanol and hexamethyldisilazane (HMDS; Sigma-Aldrich, USA) and three with 100% (v/v) HMDS (10 min each). The samples were then air-dried for 48 h, sputter-coated with platinum using a sputter coater (EM ACE200, Leica, Austria), and analyzed using FE-SEM (SIGMA, Carl Zeiss, UK) at an accelerating voltage of 5.0 kV.

### Statistical analysis

Fungal communities were analyzed using R v.4.5.0. Diversity analyses were conducted with plastic type (HDPE, LDPE, or PP) and plastisphere developmental stage (early or mature) as fixed factors. Fungal community composition was characterized, diversity metrics were calculated using the *vegan* package v.2.7.1, and plots were generated using *ggplot2* v.3.5.2 (Dixon, 2003; Wickham, 2011). For alpha diversity, the inverse Simpson and exponential Shannon indices were calculated and compared using nonparametric tests (Hill, 1973; Hurlbert, 1971; Lande, 1996). Differences in alpha diversity between developmental stages were assessed using the paired Wilcoxon signed-rank test, whereas those among plastic types were assessed using the Friedman test (Friedman, 1937; Wilcoxon, 1945). P-values were adjusted for multiple comparisons using the Benjamini–Hochberg procedure, and the adjusted p-values were reported as q-values (Benjamini and Hochberg, 1995). Beta diversity was assessed using the Jaccard index, and principal coordinates analysis (PCoA) was performed on the resulting distance matrix with the Lingoes correction to account for negative eigenvalues (Gower, 1966). Heat-tree visualizations were performed using the *metacoder* package v.0.3.8 (Foster et al., 2017).

## Results

### Visual characterization of plastisphere developmental stages

The mesocosm structures sampled at each plastisphere developmental stage showed clear differences in colonization patterns. Macroscopically, the early mesocosm structure appeared relatively clean with minimal surface colonization, whereas the mature mesocosm structure exhibited conspicuous and dense colonization by diverse marine organisms, such as macroalgae (e.g., *Ulva*, *Cladophora*, and *Pylaiella*) and sessile marine fauna, including Pacific oysters, Mediterranean mussels, barnacles, and bryozoans (Fig. 1). Microscopically, the plastic sheet surfaces exhibited increasingly complex patterns over time, consistent with the macroscopic changes. SEM observations revealed that early-stage plastic surfaces were sparsely colonized and only bacterial morphotypes were present. By contrast, mature-stage plastic sheets were thoroughly covered with rod-shaped and filamentous microorganisms, which were intricately intertwined with particulate matter. Notably, signs of surface degradation, including cracks and pitting, were observed across all plastic types in the mature stage (Fig. 2).

### Culturable fungal diversity in marine plastisphere communities

In total, 436 fungal strains were isolated from the plastic sheets sampled at the two plastisphere developmental stages: 214 and 222 strains from the early (seven days) and mature (30 days) stages, respectively. By plastic type, 132 strains were isolated from HDPE (early stage, 54; mature stage, 78), 161 from LDPE (early stage, 95; mature stage, 66), and 143 from PP (early stage, 65; mature stage, 78). ITS sequences were successfully obtained from all 436 strains, and BLAST searches identified 179 taxa, of which 178 were assigned to 57 known genera; one taxon (strain number SFC20240812-M088) remained unassigned, only possibly matching with “*Microascales* sp.” (Accession Number PZ123891). Additional genetic markers were sequenced for species-level identification, upon which 452 sequences were obtained: 23 LSU, 4 SSU, 37 *ACT*, 41 *CAM*, 5 *GAPDH*, 1 *RPB1*, 17 *RPB2*, 193 *TEF1*, and 131 *TUB2*. By using phylogenetic analyses with multi-locus datasets, 179 taxa were identified as belonging to 57 genera, 37 families, eight classes, and four phyla (data not shown).

The fungal community composition differed across the developmental stages and plastic types (Fig. 3). In the early stage, a few genera dominated the communities, whereas the diversity increased toward the mature stage. Species richness was higher in the mature stage than in the early stage, and temporal changes in species richness across the three major genera (*Penicillium*, *Cladosporium*, and *Trichoderma*) were consistent across the plastic types. *Penicillium* consistently exhibited a relatively high species richness in both stages across all plastic types, but particularly so in HDPE. *Cladosporium* showed higher species richness at the early stage across all plastic types, but decreased with maturation, whereas *Trichoderma* showed an opposite trend. *Aspergillus* and *Fusarium* also showed high species richness, with the relative richness varying across plastic types and developmental stages.

Alpha diversity indices (inverse Simpson and exponential Shannon) were higher in the mature than in the early stage (Fig. 4A). The fungal communities on PP showed higher species richness than those on HDPE and LDPE, although not significantly so. PCoA based on the Jaccard index revealed some separation in the beta diversity between developmental stages along the two main components, where the first and second principal components explained 28.9% and 20.7% of the total variance, respectively (Fig. 4B). However, no clear clustering was observed by plastic type.

### Plastic degradation assay results

Of the 436 fungal strains isolated, 238 (54.6%) showed measurable plastic degradation capacity compared to the same plastic type from which they were initially isolated. The weight-loss percentages of plastics were measured after 60 days of incubation and those of strains isolated from plastics in the early and mature stages were compared (Fig. 5). Among strains isolated from HDPE, LDPE, and PP, those from the early stage degraded plastic by 0.94–5.35%, 0.31–2.34%, and 0.30–0.90%, respectively; whereas those from the mature stage degraded plastic by 1.26–6.29%, no detectable weight loss, and 0.15–0.60% (comparable to but slightly lower than the early-stage strains), respectively. Overall, strains from the early-stage LDPE plastisphere showed a stronger degradation capacity than their mature-stage counterparts, whereas strains from the mature-stage HDPE showed a greater capacity than their early-stage counterparts. The degradation levels of the strains from both stages of the PP plastisphere were similar.

The degradation capacity varied across taxonomic classifications and plastic types (Fig. S1). Most of the genera isolated from HDPE exhibited a substantial plastic degradation capacity. The top-ranking plastic-degrading strains isolated from LDPE were from the genera *Cladosporium* and *Penicillium*, whereas some plastic-degrading strains from PP belonged to *Aspergillus*, *Nigrograna*, and *Penicillium*. The stage-level plastic degradation capacity varied across the plastic types and developmental stages (Table S3). SEM observations revealed plastic-specific and stage-dependent surface damage patterns, although visible surface disruption did not always correspond to measurable weight loss (Fig. 6). HDPE disc surfaces showed irregular cavities and fine pores when colonized by early-stage strains, and extensive cracking and lifting when colonized by mature-stage strains. LDPE disc surfaces were altered with substantial erosion, deep indentations, and increased roughness when colonized by early-stage strains, but only surface cracking occurred without apparent weight loss when colonized by mature-stage strains. PP disc surfaces showed alterations by strains from both stages (although to a lesser extent than in the cases of HDPE or LDPE) including cavities with fragmented crystalline material (by early-stage strains) and partial delamination with small pores and plate-like fragments (by mature-stage strains).

## Discussion

### Temporal progression of plastisphere development in the marine mesocosm

In this study, we investigated the culturable fungal communities in the plastispheres of three common plastic types (HDPE, LDPE, and PP) across two developmental stages (early versus mature) in a marine mesocosm experiment. Despite the relatively short deployment period in seawater, microscopic observations revealed a clear temporal progression in plastisphere development, with sparse microbial attachment in the early stage and a more complex microbial community structure in the mature stage. These trends are consistent with the known marine plastisphere developmental processes (Eich et al., 2015; Jacquin et al., 2021).

### Culturable diversity and dominant taxa of fungi colonizing marine plastispheres

Fungi were likewise found to establish dynamically on plastic surfaces during the experimental period. A total of 179 fungal taxa were isolated from the plastic sheets, indicating a substantial fungal diversity associated with marine plastispheres. Notably, a considerable number of taxa were recovered in the early developmental stage, suggesting that fungal colonization occurs rapidly following plastic exposure to marine environments. The genera *Penicillium*, *Cladosporium*, *Trichoderma*, *Aspergillus*, and *Fusarium* were dominant across all three plastic types and the two plastisphere developmental stages, which is consistent with previous studies on marine plastisphere-related fungal diversity (Philippe et al., 2023; Rakhmawati et al., 2025). These cosmopolitan saprotrophic fungi exhibit broad environmental tolerance and produce diverse extracellular enzymes (Behera and Das, 2023; Levetin et al., 2016; Ward, 2012), which likely enables their colonization of nutrient-poor plastic surfaces in dynamic marine environments. *Cladosporium* and *Penicillium* species were particularly abundant across all plastic types and developmental stages. Several of these species, such as *C. rectoides* and *P. exsudans*, have repeatedly been reported across the marine plastisphere (Florio Furno et al., 2025; Kim et al., 2022), suggesting that these genera may play important roles therein.

### Temporal shifts in species richness of dominant fungal genera

Although the overall community composition did not differ significantly between developmental stages, temporal shifts in the relative species richnesses of the dominant genera were observed. *Cladosporium* exhibited higher species richness in the early stage. Several species within this genus are known for their high osmotolerance and capacity to grow under low water activity and oligotrophic conditions (Bensch et al., 2018; Segers et al., 2015; Zalar et al., 2007). These traits may facilitate early colonization when organic resources are limited. By contrast, *Trichoderma* exhibited a higher relative species richness at the mature stage across all plastic types. *Trichoderma* species are well-known for producing various hydrolytic and oxidative enzymes, such as laccases and manganese peroxidases (Hoque et al., 2023; Othman et al., 2021; Sowmya et al., 2014). These enzymatic capabilities may be advantageous in the mature stage when microbial biomass and organic matter have accumulated on plastic surfaces.

### Environmental drivers shaping fungal community composition

Despite the abovementioned temporal trends, overall fungal community composition did not show statistically significant differences among the developmental stages or plastic types. The lack of substrate-specific clustering may reflect physicochemical properties shared by HDPE, LDPE, and PP, including high hydrophobicity and low surface energy (Encinas et al., 2010). Additionally, the close spatial arrangement of plastic sheets within mesocosm structures likely resulted in relatively homogeneous environmental conditions, reducing substrate-specific selective pressures. Other environmental factors may also have shaped the community compositions. Nutrient concentrations and stoichiometric ratios drive the succession of marine fungal communities in coastal ecosystems (Guo et al., 2015). Moreover, nutrient availability can modulate cross-kingdom interactions between bacteria and fungi, suggesting that such interactions underlie shifts in community composition (Velez et al., 2018). In accordance with this, SEM observations revealed diverse microorganisms colonizing plastic surfaces, together with clear alterations, such as increased roughness, pits, and cavities. Although these features may reflect the combined activity of multiple microbial groups, subsequent weight-loss assays demonstrated that a substantial proportion of the isolated fungal strains (54.6%) exhibited measurable degradation capacities. Furthermore, similar surface damage patterns were reproduced under controlled conditions following fungal inoculation, indicating that fungi contributed to the observed surface modifications. However, as culture-dependent approaches capture only the cultivable fraction of the fungal community, interaction-driven or fine-scale substrate-specific differences may have been underestimated. Interactions among fungi, as well as between fungi and bacteria, are also likely to influence plastisphere assembly and function through processes such as cooperative biofilm formation, nutrient exchange, and metabolic complementarity (Amaral-Zettler et al., 2020; Su et al., 2022; Wang et al., 2021). Because this study focused on culturable fungi and the degradation capacities of individual strains, these potential interactions could not be evaluated directly. Future studies using culture-independent methods, together with co-culture or functional approaches integrating fungal and bacterial community analyses, would provide a more comprehensive understanding of the dynamics, interactions, and functional roles of plastisphere communities.

### Plastic type- and stage-dependent variation in fungal degradation capacity

We initially hypothesized that mature-stage strains would exhibit a higher degradation capacity owing to the enrichment of plastic-degrading fungi during later colonization. However, the degradation capacity varied among the plastic types and developmental stages, suggesting that the contribution of fungi to plastic degradation is affected by the physicochemical properties of each plastic sheet. Consistent with our hypothesis, mature-stage strains on HDPE generally showed a higher degradation capacity. This pattern may be consistent with the time-dependent environmental filtering on HDPE surfaces, where prolonged exposure to abiotic weathering (e.g., UV, salinity, and mechanical abrasion) increases surface oxidation and roughness, thereby improving microbial attachment and enzymatic accessibility relative to freshly immersed HDPE (Ghatge et al., 2020; Montazer et al., 2020). By contrast, LDPE showed relatively high degradation by early-stage strains, suggesting that opportunistic early colonizers with high degradation capacity exploit the more accessible physicochemical structure of LDPE, thereby leaving fewer opportunities for later degraders (Mohanan et al., 2020; Pinto et al., 2020). The PP degradation capacity was comparable between strains from each developmental stage, possibly because of the higher crystallinity and hydrophobicity of PP compared to those of HDPE and LDPE, which impede microbial attachment and enzyme access (Choonut et al., 2025; Guo et al., 2024; Srikanth et al., 2022) and may limit degradation regardless of the plastisphere developmental stage.

### Taxonomic patterns and functional heterogeneity of plastisphere fungi

Species with high degradation capacities were unevenly distributed across genera; however, some trends in genus-level compositional changes were observed. The strains exhibiting the highest degradation capacity belonged to *Aspergillus*, *Penicillium*, *Cladosporium*, and *Trichoderma*, which have previously been reported as effective plastic degraders (Kim et al., 2022; Ma et al., 2025; Zeghal et al., 2021). Notably, the observed fungal degradation capacity did not show a strong association with plastic type or developmental stage, suggesting that species-level functional traits are more important determinants of degradation performance than factors depending on the environments from which strains were isolated. However, 45.4% of the strains exhibited no detectable plastic degradation. These non-degrading strains likely fulfilled alternative ecological roles within plastisphere communities. Some may have utilized organic matter derived from other marine organisms, contributing to nutrient cycling rather than direct plastic degradation (Peng et al., 2024; Salazar-Alekseyeva et al., 2023; Wang et al., 2016). Others may have contributed to biofilm formation by producing EPS, thereby enhancing structural stability and facilitating the adhesion of other organisms (Decho and Gutierrez, 2017; Qi et al., 2022). Together, these findings suggest that fungi contribute to plastisphere functioning in diverse ways beyond direct plastic degradation, including biofilm formation, structural stabilization, and nutrient cycling, thereby sustaining the plastisphere as a microecosystem.

## Conclusions and Future Perspectives

This study provides novel insights into the composition and functional capacities of culturable fungal communities in the marine plastisphere across different plastic types and developmental stages. Using a cultivation-based approach, we directly assessed plastic degradation at the taxonomic level and found that the degradation potential varied among species, with no consistent relationship between the plastic type or plastisphere developmental stage and degradation capacity. Furthermore, the detection of both degrading and non-degrading fungi indicated the functional heterogeneity of plastisphere communities, suggesting that non-degraders may contribute to other ecological processes, such as nutrient cycling and biofilm structural stability. In addition, the fungal strains identified as active degraders are promising candidates for future applications in plastic bioremediation. Future studies integrating culture-independent approaches with finer temporal sampling over long incubation periods will complement the present findings and further refine our understanding of fungal succession and the ecological role of fungi in marine plastisphere.

## Figures and Tables

**Fig. 1.**
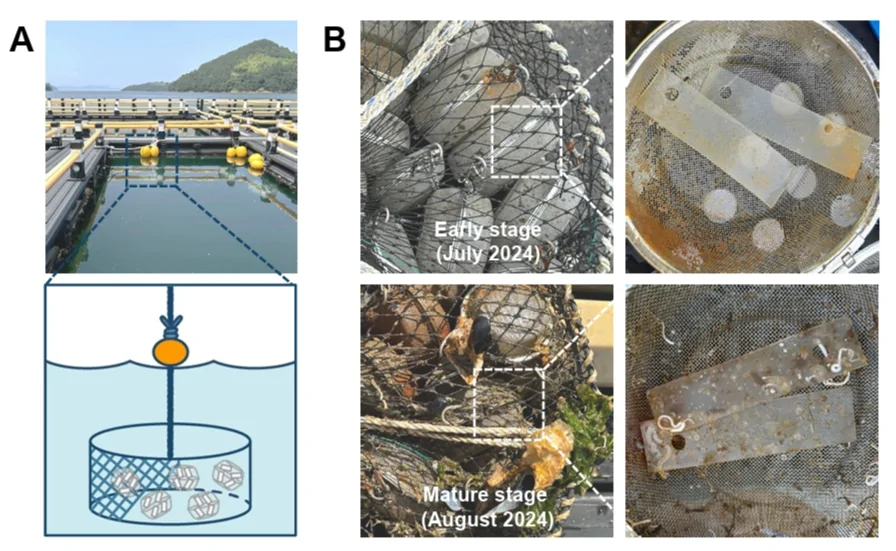
Mesocosm experimental design and field photographs of plastic sheets across plastisphere developmental stages. (A) Photograph of the research site where the mesocosm experiment was conducted (top) and schematic illustration of a mesocosm structure (bottom). (B) Photographs of plastic sheets showing surface colonization patterns in the two plastisphere developmental stages; top panels: early stage (seven days after deployment, July 2024); bottom panels: mature stage (30 days after deployment, August 2024).

**Fig. 2.**
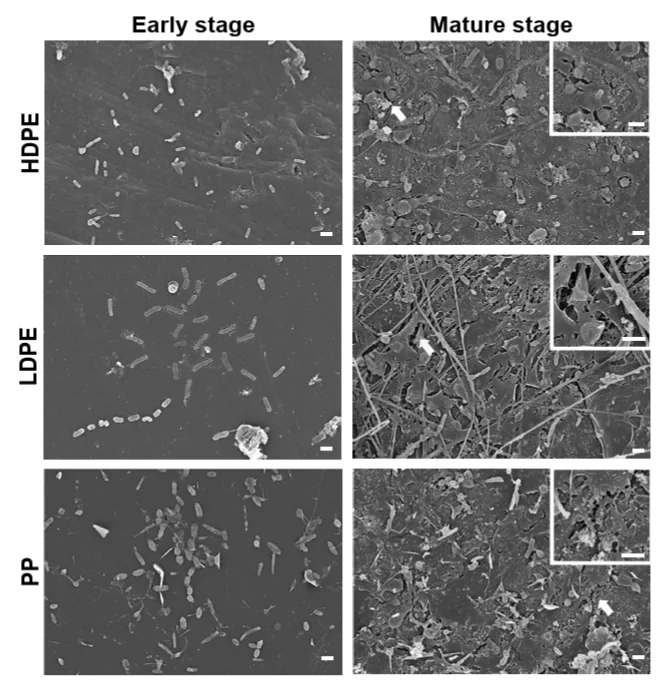
Scanning electron micrographs of plastic sheet surfaces from the mesocosm experiment. Representative SEM images of high-density polyethylene (HDPE; top), low-density polyethylene (LDPE; middle), and polypropylene (PP; bottom) surfaces sampled at the early (left) and mature (right) stages. Insets in the mature-stage micrographs show higher-magnification views of the regions indicated by arrows, highlighting surface morphology and microbial colonization. Scale bars = 1 μm.

**Fig. 3.**
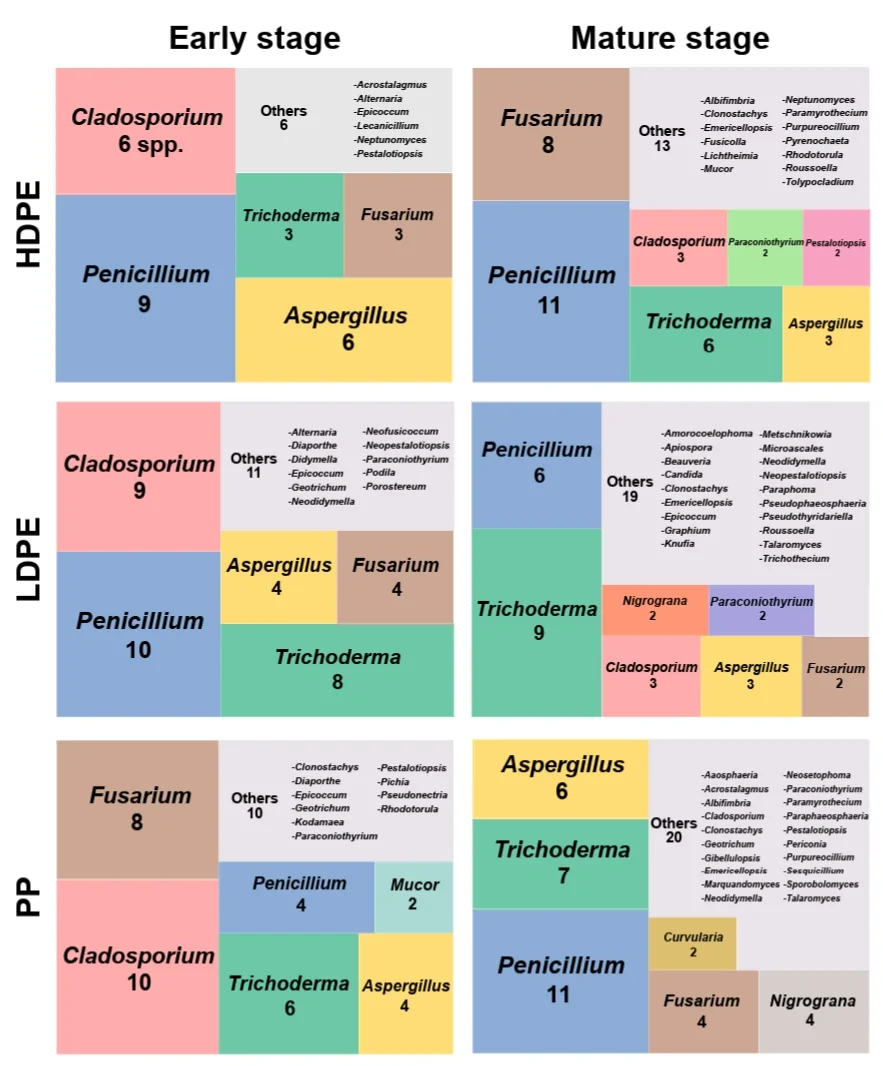
Culturable fungal communities across plastic types and developmental stages. Treemaps show the relative species richness of fungal genera isolated from high-density polyethylene (HDPE; top), low-density polyethylene (LDPE; middle), and polypropylene (PP; bottom) in the early (left) and mature (right) stages. Box sizes represent the relative number of species within each genus, and numbers indicate the number of species per genus. “Others” represent genera from which only a single species was present; the number indicates the total count of such genera, with individual genus names listed alongside.

**Fig. 4.**
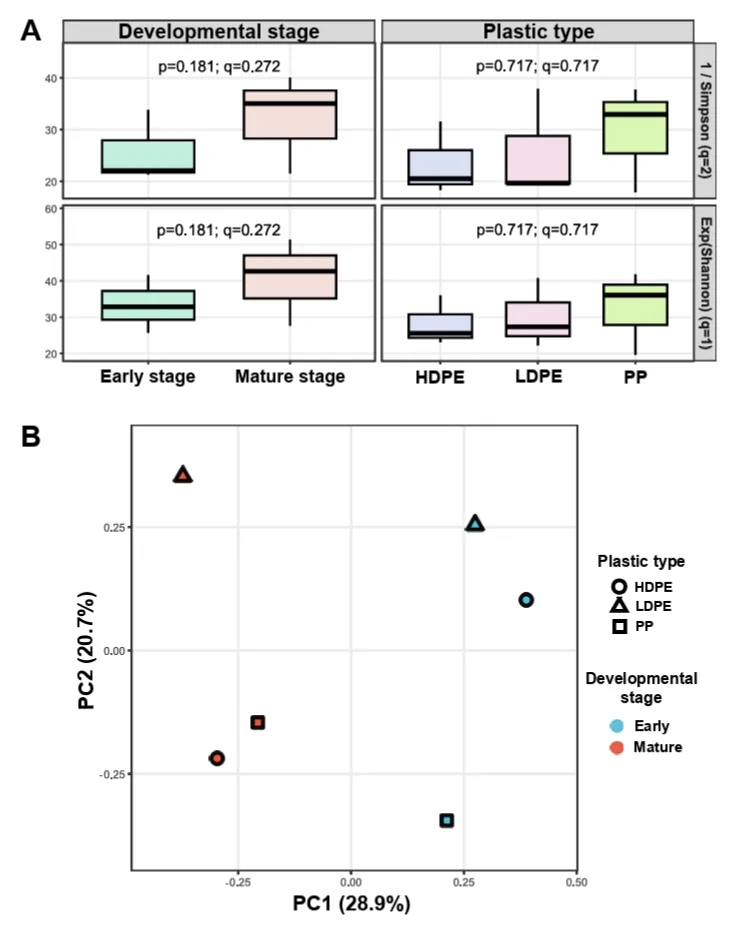
Alpha and beta diversity of culturable fungal communities across plastic types and developmental stages. (A) Alpha diversity metrics compared among plastic types (high-density polyethylene [HDPE], low-density polyethylene [LDPE], polypropylene [PP]) and developmental stages (early and mature). Top row: inverse Simpson diversity (q = 2); bottom row: exponential Shannon diversity (q = 1). Boxes show medians and interquartile ranges, and reported p- and q-values from statistical tests are indicated. (B) Beta diversity based on Jaccard similarity visualized by principal coordinates analysis. The first (PC1) and second (PC2) principal components explain 28.9% and 20.7% of the variance, respectively. Point indicators are shaped by plastic type and colored by developmental stage.

**Fig. 5.**
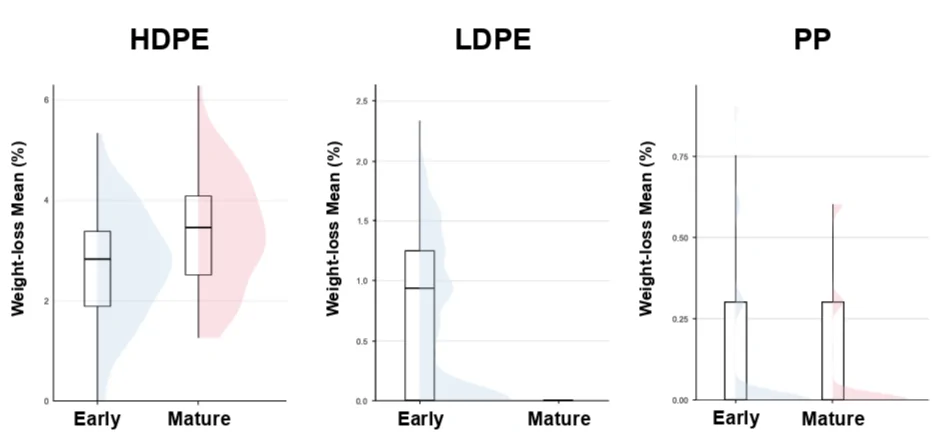
Degradation performance across isolated fungal strains according to plastic type and developmental origin. Violin plots show the distribution of weight loss percentages after 60 days of incubation with fungal strains tested against the same plastic type from which they were originally isolated. Strains are grouped by the developmental stage of the source plastisphere: early (blue) and mature (pink). Boxplots indicate medians and interquartile ranges. Panels show results for strains from the high-density polyethylene (left), low-density polyethylene (middle), and polypropylene (right) plastispheres.

**Fig. 6.**
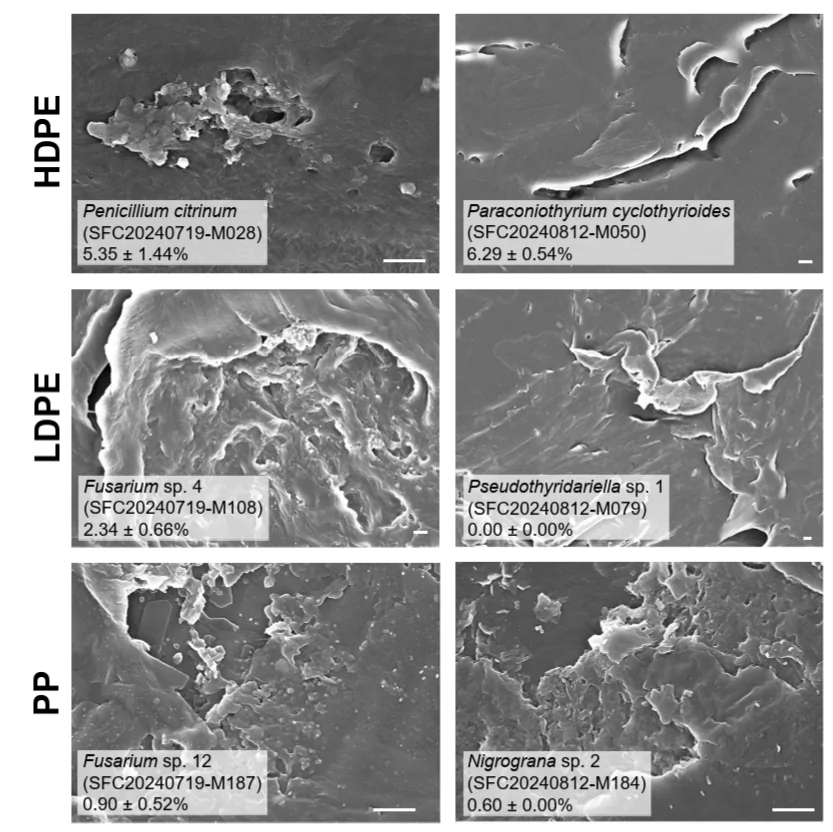
Surface morphology of plastic discs after 60-day fungal degradation assays. SEM images show plastic surfaces incubated for 60 days with fungal strains exhibiting the highest degradation capacity for each plastisphere type. Left panels: plastics inoculated with strains from the early stage; right panels: plastics inoculated with strains from the mature stage. Top row: high-density polyethylene (HDPE); middle row: low-density polyethylene (LDPE); bottom row: polypropylene (PP). Scale bars = 1 μm.

## Supplementary Information

Table S1. Information on the primer sets used in PCR, the PCR conditions, and the genera to which they were applied

Table S2. Representative strain information and GenBank accessions used in this study

Table S3. Top plastic-degrading fungal strains identified in this study. Weight-loss (%) values are shown as Means ± standard deviations for each plastic type and exposure time.

Fig. S1. Taxonomic distribution of plastic degradation ability across fungal strains. Heat trees show hierarchical taxonomic relationships and mean weight-loss assay results (%) of fungal strains isolated from (A) HDPE, (B) LDPE, and (C) PP. Each terminal node represents a fungal species, and node size reflects the number of strains within each taxonomic group. Node color intensity represents the mean value of weight-loss assay results (weight loss %).
