# Supplementary material for: Temporal changes in culturable fungal community composition with their plastic degradation capacities in the marine plastisphere through mesocosm experiments
Source: J Microbiol. 2026 Aug 31;64(8):e2605011. doi: 10.71150/jm.2605011 (PMC13688127; doi:10.71150/jm.2605011)
Supplement: Supplementary file 1 [file jm-2605011-Supplementary-Fig-S1.pdf]

A

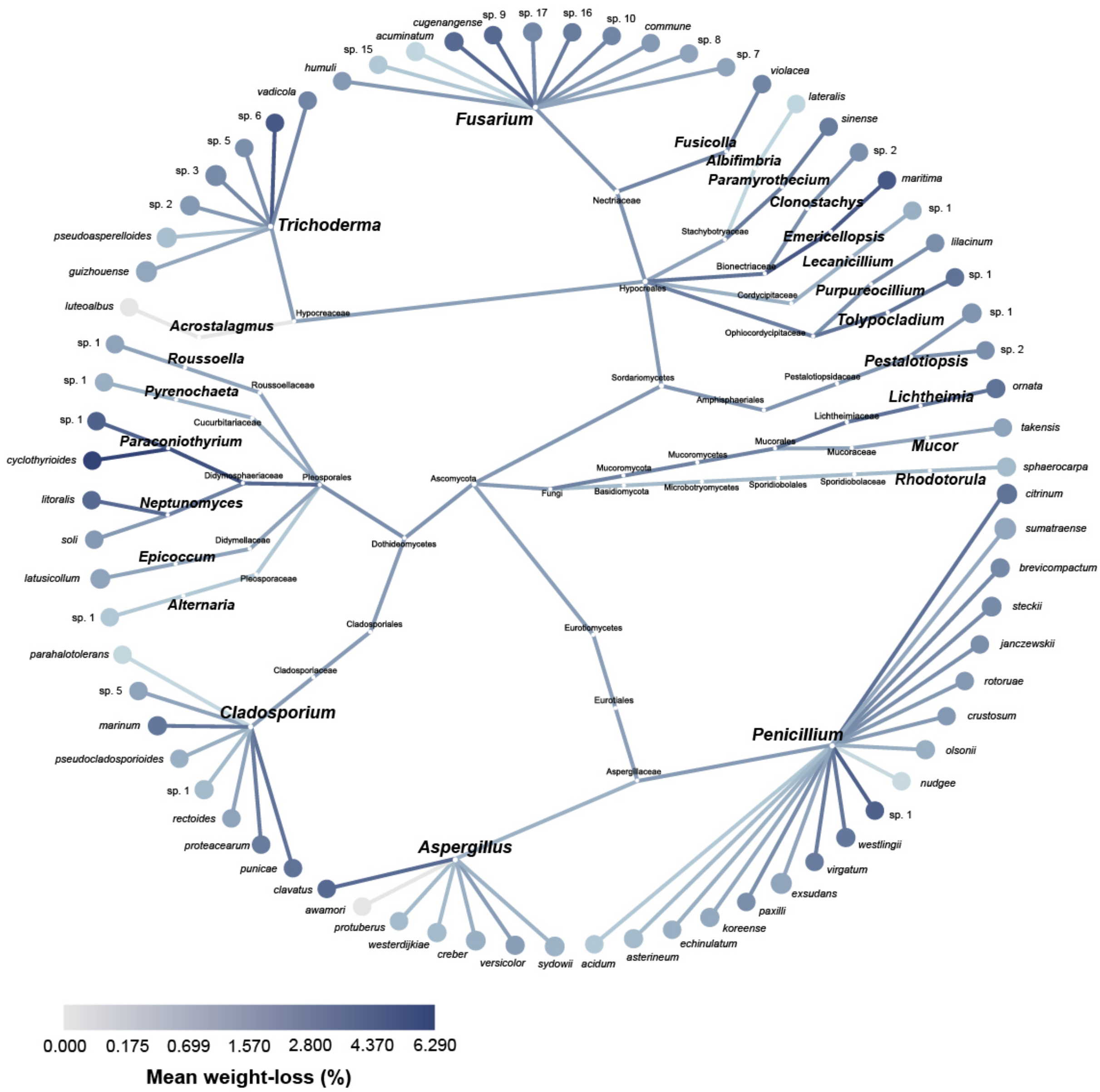

Fig. S1. Taxonomic distribution of plastic degradation ability across fungal strains. Heat trees show hierarchical taxonomic relationships and mean weight-loss assay results (%) of fungal strains isolated from (A) HDPE, (B) LDPE, and (C) PP. Each terminal node represents a fungal species, and node size reflects the number of strains within each taxonomic group. Node color intensity represents the mean value of weight-loss assay results (weight loss %).

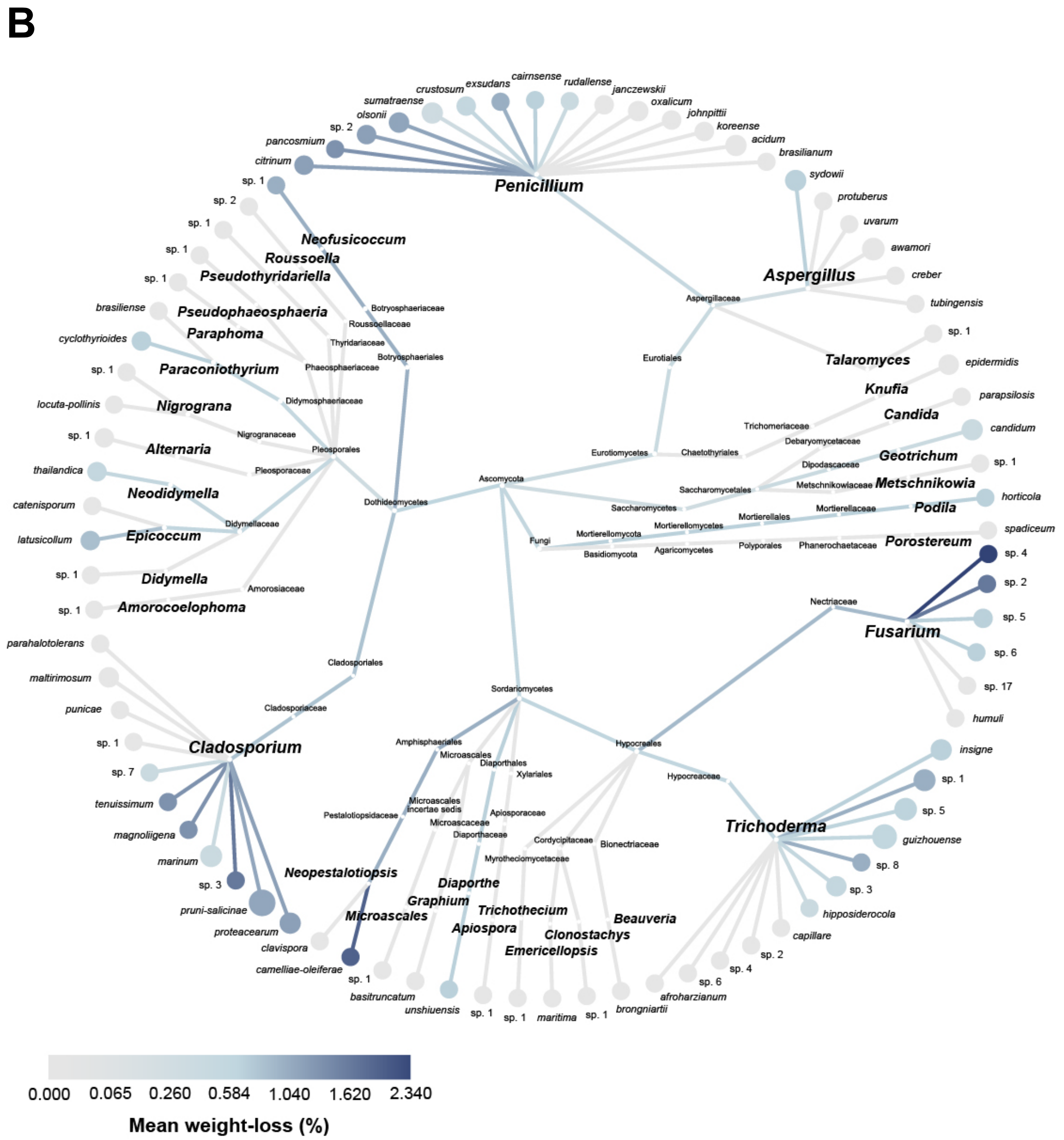

Fig. S1. (Continued)

C

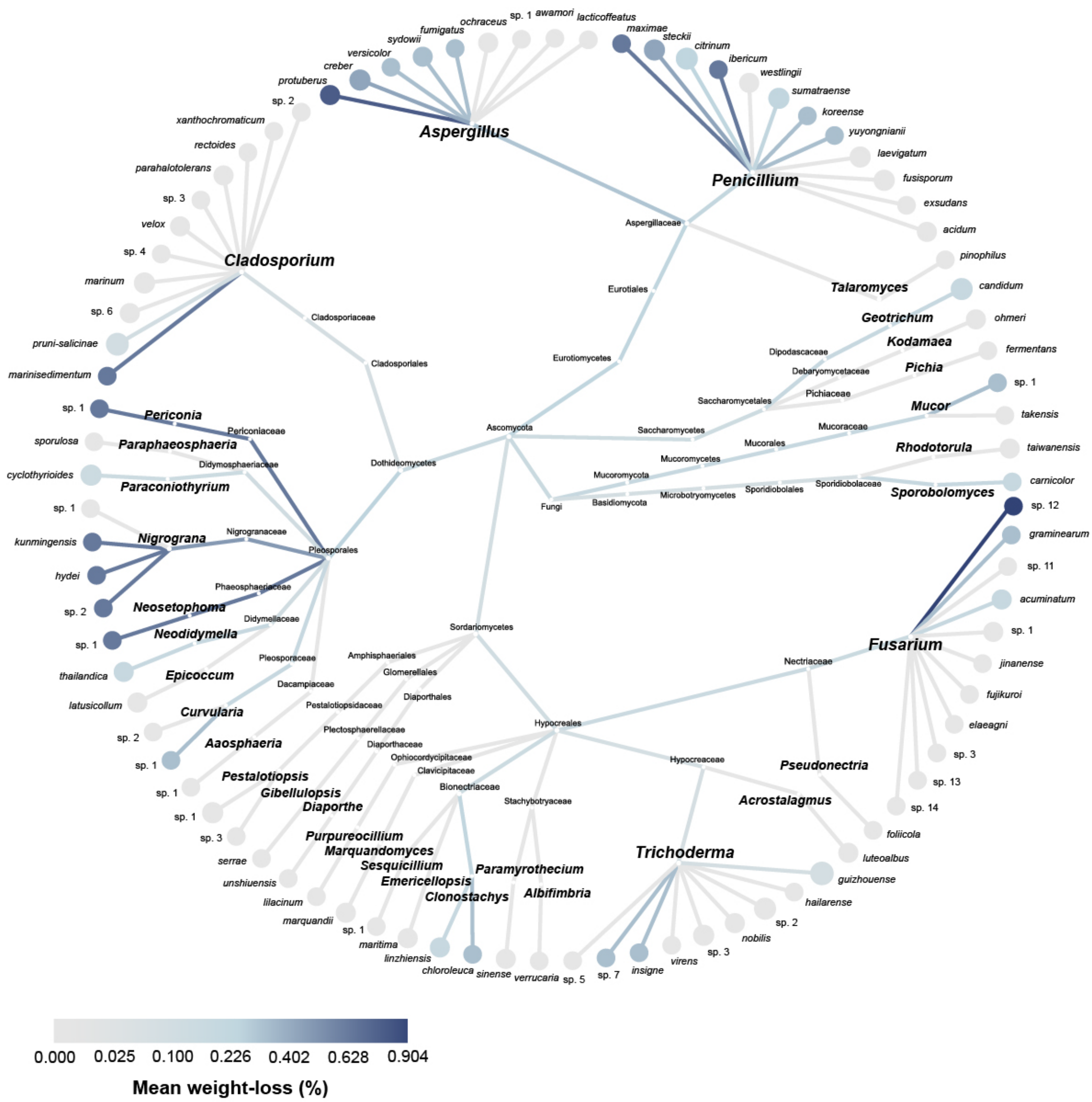

Fig. S1. (Continued)
