# Supplementary material for: Temporal changes in culturable fungal community composition with their plastic degradation capacities in the marine plastisphere through mesocosm experiments
Source: J Microbiol. 2026 Aug 31;64(8):e2605011. doi: 10.71150/jm.2605011 (PMC13688127; doi:10.71150/jm.2605011)
Supplement: Supplementary file 2 [file jm-2605011-Supplementary-Table-S2.pdf]

Table S2. Representative strain information and GenBank accessions used in this study

Species	Strain	Plastictype	ITS	LSU	SSU	*ACT*	*CAM*	*RPB1*	*RPB2*	*TEF1*	*TUB2*	*GAPDH*
Aaosphaeria sp. 1	SFC20240812-M154	PP	PZ123936									
Acrostalagmus luteoalbus	SFC20240719-M005	HDPE	PZ123785									
Albifimbria lateralis	SFC20240812-M032	HDPE	PZ123825							PZ136128		
Albifimbria verrucaria	SFC20240812-M174	PP	PZ123941							PZ136129		
Alternaria sp. 1	SFC20240719-M010	HDPE	PZ123788						PZ136127	PZ136126		PZ136130
Amorocoelophoma sp. 1	SFC20240812-M086	LDPE	PZ123889	PZ162092	PZ162091				PZ136131	PZ136132		
Apiospora sp. 1	SFC20240812-M098	LDPE	PZ123896								PZ136133	
Aspergillus awamori	SFC20240719-M147	LDPE	PZ123881				PZ136141					
Aspergillus clavatus	SFC20240812-M048	HDPE	PZ123840				PZ136137					
Aspergillus creber	SFC20240719-M032	HDPE	PZ123798									
Aspergillus fumigatus	SFC20240812-M197	PP	PZ123951				PZ136145					
Aspergillus lacticoffeatus	SFC20240812-M148	PP	PZ123934				PZ136140					
Aspergillus ochraceus	SFC20240812-M161	PP	PZ123938				PZ136144					
Aspergillus protuberus	SFC20240719-M047	HDPE	PZ123808				PZ136135					
Aspergillus sp. 1	SFC20240812-M147	PP	PZ123933				PZ136143					
Aspergillus sydowii	SFC20240719-M048	HDPE	PZ123809				PZ136136					
Aspergillus tubingensis	SFC20240812-M096	LDPE	PZ123894				PZ136139					
Aspergillus uvarum	SFC20240812-M095	LDPE	PZ123893				PZ136138					
Aspergillus versicolor	SFC20240719-M027	HDPE	PZ123796				PZ136134					
Aspergillus westerdijkiae	SFC20240719-M006	HDPE	PZ123786				PZ136142					
Beauveria brongniartii	SFC20240812-M099	LDPE	PZ123897					PZ197393		PZ136123		
Candida parapsilosis	SFC20240812-M081	LDPE	PZ123884	PZ159300								
Cladosporium magnoliigena	SFC20240719-M116	LDPE	PZ123877			PZ166936						
Cladosporium maltirimosum	SFC20240812-M126	LDPE	PZ123903			PZ166935				PZ161603		
Cladosporium marinisedimentum	SFC20240812-M190	PP	PZ123949							PZ166942		
Cladosporium marinum	SFC20240812-M026	HDPE	PZ123822							PZ170378		
Cladosporium parahalotolerans	SFC20240812-M028	HDPE	PZ123823			PZ166937						
Cladosporium proteacearum	SFC20240719-M022	HDPE	PZ123794							PZ170379		

Cladosporium pruni-salicina	SFC20240719-M020	HDPE	PZ123793	PZ197394		PZ170380	
Cladosporium pseudocladosporioides	SFC20240719-M018	HDPE	PZ123792	PZ166938		PZ170381	
Cladosporium punicae	SFC20240719-M014	HDPE	PZ123789			PZ166943	
Cladosporium rectoides	SFC20240719-M015	HDPE	PZ123790			PZ170382	
Cladosporium sp. 1	SFC20240719-M016	HDPE	PZ123791			PZ170375	
Cladosporium sp. 2	SFC20240719-M165	PP	PZ123916				
Cladosporium sp. 3	SFC20240719-M129	LDPE	PZ123878			PZ170376	
Cladosporium sp. 4	SFC20240719-M172	PP	PZ123917			PZ170377	
Cladosporium sp. 5	SFC20240812-M025	HDPE	PZ123821			PZ170383	
Cladosporium sp. *6*	SFC20240719-M174	PP	PZ123918	PZ166939		PZ170384	
Cladosporium sp. *7*	SFC20240719-M114	LDPE	PZ123876	PZ166940		PZ170385	
Cladosporium tenuissimum	SFC20240719-M131	LDPE	PZ123879	PZ166941		PZ170386	
Cladosporium velox	SFC20240719-M178	PP	PZ123919			PZ166944	
Cladosporium xanthochromaticum	SFC20240719-M164	PP	PZ123915			PZ166945	
Clonostachys chloroleuca	SFC20240719-M212	PP	PZ123930				
Clonostachys linzhiensis	SFC20240812-M193	PP	PZ123950				
Clonostachys sp. 1	SFC20240812-M097	LDPE	PZ123895				PZ158588
Clonostachys sp. 2	SFC20240812-M039	HDPE	PZ123832				
Curvularia sp. 1	SFC20240812-M189	PP	PZ123948				
Curvularia sp. 2	SFC20240812-M215	PP	PZ123957				
Diaporthe unshiuensis	SFC20240719-M085	LDPE	PZ123865				PZ158589
Didymella sp. 1	SFC20240719-M141	LDPE	PZ123880				
Emericellopsis maritima	SFC20240812-M030	HDPE	PZ123824		PZ158536	PZ158554	
Epicoccum catenisporum	SFC20240812-M103	LDPE	PZ123899				PZ158591
Epicoccum latusicollum	SFC20240719-M009	HDPE	PZ123787		PZ158537		PZ158590
Fusarium acuminatum	SFC20240719-M002	HDPE	PZ123784			PZ158555	
Fusarium commune	SFC20240812-M042	HDPE	PZ123835			PZ155120	
Fusarium cugenangense	SFC20240812-M033	HDPE	PZ123826			PZ158556	
Fusarium elaeagni	SFC20240719-M210	PP	PZ123929			PZ155127	
Fusarium fujikuroi	SFC20240719-M213	PP	PZ123931			PZ155128	
Fusarium graminearum	SFC20240719-M185	PP	PZ123921			PZ155125	

Fusarium humuli	SFC20240719-M044	HDPE	PZ123805		PZ158557
Fusarium jinanense	SFC20240719-M154	PP	PZ123911		PZ155124
Fusarium sp. 1	SFC20240719-M204	PP	PZ123926		PZ155126
Fusarium sp. 10	SFC20240812-M044	HDPE	PZ123837		
Fusarium sp. 11	SFC20240719-M186	PP	PZ123922		
Fusarium sp. 12	SFC20240719-M187	PP	PZ123923		
Fusarium sp. 13	SFC20240812-M167	PP	PZ123939		
Fusarium sp. 14	SFC20240812-M220	PP	PZ123960		
Fusarium sp. 15	SFC20240719-M045	HDPE	PZ123806		
Fusarium sp. 16	SFC20240812-M034	HDPE	PZ123827		
Fusarium sp. 17	SFC20240812-M041	HDPE	PZ123834		PZ158558
Fusarium sp. 2	SFC20240719-M110	LDPE	PZ123873		
Fusarium sp. 3	SFC20240812-M170	PP	PZ123940		PZ155129
Fusarium sp. 4	SFC20240719-M108	LDPE	PZ123871		PZ155123
Fusarium sp. 5	SFC20240719-M057	LDPE	PZ123857		PZ155121
Fusarium sp. 6	SFC20240719-M079	LDPE	PZ123862		PZ155122
Fusarium sp. 7	SFC20240812-M037	HDPE	PZ123830		
Fusarium sp. 8	SFC20240812-M036	HDPE	PZ123829		
Fusarium sp. 9	SFC20240812-M043	HDPE	PZ123836		
Fusicolla violacea	SFC20240812-M074	HDPE	PZ123853		
Geotrichum candidum	SFC20240719-M082	LDPE	PZ123863	PZ146638	
Gibellulopsis serrae	SFC20240812-M212	PP	PZ123955	PZ146723	
Graphium basitruncatum	SFC20240812-M109	LDPE	PZ123900		
Knufia epidermidis	SFC20240812-M090	LDPE	PZ123892		
Kodamaea ohmeri	SFC20240719-M184	PP	PZ123920		
Lecanicillium sp. 1	SFC20240719-M001	HDPE	PZ123783		PZ136125
Lichtheimia ornata	SFC20240812-M053	HDPE	PZ123844		
Marquandomyces marquandii	SFC20240812-M151	PP	PZ123935		
Metschnikowia sp. 1	SFC20240812-M082	LDPE	PZ123885		
Microascales sp. 1	SFC20240812-M088	LDPE	PZ123891		
Mucor sp. 1	SFC20240719-M189	PP	PZ123924	PZ158381	
Mucor takensis	SFC20240812-M051	HDPE	PZ123843	PZ158380	

Neodidymella thailandica	SFC20240719-M113	LDPE	PZ123875								
Neofusicoccum sp. 1	SFC20240719-M111	LDPE	PZ123874							PZ158559	
Neopestalotiopsis camelliae-oleiferae	SFC20240719-M107	LDPE	PZ123870							PZ158560	PZ158592
Neopestalotiopsis clavispora	SFC20240812-M141	LDPE	PZ123907							PZ158561	PZ158593
Neosetophoma sp.1	SFC20240812-M213	PP	PZ123956								
Neptunomyces litoralis	SFC20240812-M049	HDPE	PZ123841								
Neptunomyces soli	SFC20240719-M054	HDPE	PZ123811	PZ146853	PZ146866				PZ158538	PZ158562	
Nigrograna hydei	SFC20240812-M185	PP	PZ123945							PZ158566	
Nigrograna kunmingensis	SFC20240812-M186	PP	PZ123946							PZ158567	
Nigrograna locuta-pollinis	SFC20240812-M087	LDPE	PZ123890							PZ158563	
Nigrograna sp. 1	SFC20240812-M124	LDPE	PZ123902							PZ158565	
Nigrograna sp. 2	SFC20240812-M184	PP	PZ123944							PZ158564	
Paraconiothyrium brasiliense	SFC20240812-M083	LDPE	PZ123886								
Paraconiothyrium cyclothyrioides	SFC20240812-M050	HDPE	PZ123842								PZ158594
Paraconiothyrium sp. 1	SFC20240812-M058	HDPE	PZ123846								PZ158595
Paramyrothecium sinense	SFC20240812-M040	HDPE	PZ123833								PZ158596
Paraphaeosphaeria sporulosa	SFC20240812-M179	PP	PZ123943								
Paraphoma sp. 1	SFC20240812-M080	LDPE	PZ123883								
Penicillium acidum	SFC20240812-M006	HDPE	PZ123813								PZ158597
Penicillium asterineum	SFC20240812-M013	HDPE	PZ123815								PZ158598
Penicillium brasilianum	SFC20240812-M116	LDPE	PZ123901								PZ158599
Penicillium brevicompactum	SFC20240719-M038	HDPE	PZ123801								PZ158600
Penicillium cairnsense	SFC20240719-M069	LDPE	PZ123859								PZ158601
Penicillium citrinum	SFC20240719-M028	HDPE	PZ123797								PZ158602
Penicillium crustosum	SFC20240719-M041	HDPE	PZ123803								PZ158603
Penicillium echinulatum	SFC20240812-M005	HDPE	PZ123812								PZ158604
Penicillium exsudans	SFC20240812-M019	HDPE	PZ123817								PZ158605
Penicillium fusisporum	SFC20250408-M001	PP	PV707061								PV737705
Penicillium ibericum	SFC20240719-M198	PP	PZ123925								PZ158606
Penicillium janczewskii	SFC20240719-M046	HDPE	PZ123807								PZ158607
Penicillium johnpittii	SFC20240812-M128	LDPE	PZ123904								PZ158608

Penicillium koreense	SFC20240812-M038	HDPE	PZ123831								PZ158609
Penicillium laevigatum	SFC20240812-M178	PP	PZ123942								PZ158610
Penicillium maximae	SFC20240812-M199	PP	PZ123953								PZ158611
Penicillium nudgee	SFC20250408-M004	HDPE	PV707064				PV737712				PV737708
Penicillium olsonii	SFC20240719-M042	HDPE	PZ123804								PZ158612
Penicillium oxalicum	SFC20240812-M143	LDPE	PZ123908								PZ197395
Penicillium pancosmium	SFC20240719-M056	LDPE	PZ123856								PZ158613
Penicillium paxilli	SFC20240812-M012	HDPE	PZ123814								PZ158614
Penicillium rotoruae	SFC20240719-M036	HDPE	PZ123800								PZ158615
Penicillium rudallense	SFC20240719-M074	LDPE	PZ123860								PZ158616
Penicillium sp. 1	SFC20240812-M022	HDPE	PZ123818				PZ136124				PZ158617
Penicillium sp. 2	SFC20240719-M058	LDPE	PZ123858								PZ158618
Penicillium steckii	SFC20240719-M033	HDPE	PZ123799								PZ158619
Penicillium sumatraense	SFC20240719-M039	HDPE	PZ123802								PZ158620
Penicillium taii	SFC20250408-M006	PP	PV707066								PV737710
Penicillium virgatum	SFC20240812-M018	HDPE	PZ123816								PZ158621
Penicillium westlingii	SFC20240812-M047	HDPE	PZ123839								PZ158622
Periconia sp. 1	SFC20240812-M187	PP	PZ123947	PZ162087							
Pestalotiopsis sp. 1	SFC20240812-M064	HDPE	PZ123848							PZ158568	PZ158623
Pestalotiopsis sp. 2	SFC20240812-M035	HDPE	PZ123828							PZ158569	PZ158624
Pestalotiopsis sp. 3	SFC20240719-M208	PP	PZ123928								PZ158625
Pichia fermentans	SFC20240719-M150	PP	PZ123909								
Podila horticola	SFC20240719-M077	LDPE	PZ123861								
Porostereum spadiceum	SFC20240719-M083	LDPE	PZ123864								
Pseudonectria foliicola	SFC20240719-M205	PP	PZ123927								
Pseudophaeosphaeria sp. 1	SFC20240812-M084	LDPE	PZ123887								
Pseudothyridariella sp. 1	SFC20240812-M079	LDPE	PZ123882								
Purpureocillium lilacinum	SFC20240812-M077	HDPE	PZ123855							PZ158570	
Pyrenochaeta sp. 1	SFC20240812-M023	HDPE	PZ123819	PZ162086	PZ162089				PZ161604	PZ161602	
Rhodotorula sphaerocarpa	SFC20240812-M076	HDPE	PZ123854	PZ158849							
Rhodotorula taiwanensis	SFC20240719-M152	PP	PZ123910	PZ158850							
Roussoella sp. 1	SFC20240812-M024	HDPE	PZ123820								

Roussoella sp. 2	SFC20240812-M106	LDPE	N/A	PZ162090	PZ162088				PZ161605	PZ166946	
Sesquicillium sp. 1	SFC20240812-M155	PP	PZ123937								
Sporobolomyces carnicolor	SFC20240812-M145	PP	PZ123932								
Talaromyces pinophilus	SFC20240812-M216	PP	PZ123958				PZ170435				PZ170436
Talaromyces sp. 1	SFC20240812-M085	LDPE	PZ123888				PZ161601				PZ170437
Tolypocladium sp. 1	SFC20240812-M046	HDPE	PZ123838								
Trichoderma afroharzianum	SFC20240812-M134	LDPE	PZ123905							PZ136118	
Trichoderma capillare	SFC20240719-M092	LDPE	PZ123867							PZ136122	
Trichoderma guizhouense	SFC20240812-M071	HDPE	PZ123852							PZ136116	
Trichoderma hailarense	SFC20240719-M159	PP	PZ123914								
Trichoderma hipposiderocola	SFC20240719-M098	LDPE	PZ123869							PZ136109	
Trichoderma insigne	SFC20240719-M109	LDPE	PZ123872							PZ136112	
Trichoderma nobilis	SFC20240719-M155	PP	PZ123912							PZ136110	
Trichoderma pseudoasperelloides	SFC20240812-M066	HDPE	PZ123850							PZ136111	
Trichoderma sp. 1	SFC20240719-M089	LDPE	PZ123866								
Trichoderma sp. 2	SFC20240812-M065	HDPE	PZ123849							PZ136121	
Trichoderma sp. 3	SFC20240812-M069	HDPE	PZ123851							PZ136117	
Trichoderma sp. 4	SFC20240812-M135	LDPE	PZ123906							PZ136114	
Trichoderma sp. 5	SFC20240812-M057	HDPE	PZ123845							PZ136113	
Trichoderma sp. 6	SFC20240812-M063	HDPE	PZ123847							PZ136115	
Trichoderma sp. 7	SFC20240812-M219	PP	PZ123959							PZ136119	
Trichoderma sp. 8	SFC20240719-M094	LDPE	PZ123868							PZ171126	
Trichoderma vadicola	SFC20240719-M053	HDPE	PZ123810							PZ136108	
Trichoderma virens	SFC20240719-M157	PP	PZ123913							PZ136120	
Trichothecium sp. 1	SFC20240812-M102	LDPE	PZ123898	PZ162093							
