# Supplementary material for: Temporal changes in culturable fungal community composition with their plastic degradation capacities in the marine plastisphere through mesocosm experiments
Source: J Microbiol. 2026 Aug 31;64(8):e2605011. doi: 10.71150/jm.2605011 (PMC13688127; doi:10.71150/jm.2605011)
Supplement: Supplementary file 3 [file jm-2605011-Supplementary-Table-S3.pdf]

Table S3. Top plastic-degrading fungal strains identified in this study. Weight-loss (%) values are shown as Means ± standard deviations for each plastic type and exposure time

Plastic type	Stage	Species	ID	Weight-loss (%)
HDPE	Early	*Penicillium citrinum*	SFC20240719-M028	5.35 ± 1.44
		Penicillium sumatraense	SFC20240719-M039	4.40 ± 0.54
		Cladosporium punicae	SFC20240719-M014	4.40 ± 0.54
		Penicillium brevicompactum	SFC20240719-M038	4.09 ± 1.44
		Penicillium steckii	SFC20240719-M033	4.09 ± 1.44
		Penicillium citrinum	SFC20240719-M035	4.09 ± 1.09
		Cladosporium proteacearum	SFC20240719-M022	4.09 ± 1.44
		Penicillium steckii	SFC20240719-M037	4.09 ± 0.54
	Mature	*Paraconiothyrium cyclothyrioides*	SFC20240812-M050	6.29 ± 0.54
		Trichoderma sp. 6	SFC20240812-M063	5.35 ± 1.09
		Emericellopsis maritima	SFC20240812-M030	5.35 ± 1.44
		Fusarium cugenangense	SFC20240812-M033	5.03 ± 1.44
		Trichoderma sp. 3	SFC20240812-M069	5.03 ± 1.44
		Penicillium sp. 1	SFC20240812-M022	5.03 ± 1.96
		Paraconiothyrium sp. 1	SFC20240812-M058	5.03 ± 1.44
LDPE	Early	*Fusarium* sp. 4	SFC20240719-M108	2.34 ± 0.66
		Cladosporium proteacearum	SFC20240719-M122	1.87 ± 0.93
		Cladosporium pruni-salicina	SFC20240719-M133	1.87 ± 0.93
		Neopestalotiopsis camelliae-oleiferae	SFC20240719-M107	1.87 ± 0.93
		Cladosporium pruni-salicina	SFC20240719-M125	1.87 ± 0.93
		Cladosporium sp. 3	SFC20240719-M129	1.56 ± 0.54
		Epicoccum latusicollum	SFC20240719-M086	1.56 ± 0.54
		Penicillium citrinum	SFC20240719-M070	1.56 ± 1.43
		Trichoderma insigne	SFC20240719-M109	1.56 ± 0.54
		Trichoderma sp. 1	SFC20240719-M089	1.56 ± 1.43
		Trichoderma sp. 1	SFC20240719-M105	1.56 ± 0.54
		Cladosporium pruni-salicina	SFC20240719-M112	1.56 ± 0.54
		Cladosporium marinum	SFC20240719-M132	1.56 ± 0.54
		Cladosporium pruni-salicina	SFC20240719-M127	1.56 ± 1.08
		Cladosporium pruni-salicina	SFC20240719-M130	1.56 ± 0.54
		Fusarium sp. 2	SFC20240719-M110	1.56 ± 1.43
PP	Early	*Fusarium* sp. 12	SFC20240719-M187	0.90 ± 0.52
		Aspergillus protuberus	SFC20240719-M195	0.90 ± 0.52
		Aspergillus creber	SFC20240719-M194	0.60 ± 0.00
		Aspergillus protuberus	SFC20240719-M196	0.60 ± 0.00
		Penicillium steckii	SFC20240719-M199	0.60 ± 0.00

	Penicillium citrinum	SFC20240719-M193	0.60 ± 0.00
	Penicillium ibericum	SFC20240719-M198	0.60 ± 0.00
	Penicillium steckii	SFC20240719-M201	0.60 ± 0.00
	Cladosporium pruni-salicina	SFC20240719-M176	0.30 ± 0.52
	Fusarium graminearum	SFC20240719-M185	0.30 ± 0.52
	Mucor sp. 1	SFC20240719-M189	0.30 ± 0.52
	Aspergillus versicolor	SFC20240719-M192	0.30 ± 0.52
	Aspergillus creber	SFC20240719-M197	0.30 ± 0.52
	Penicillium citrinum	SFC20240719-M200	0.30 ± 0.52
	Aspergillus sydowii	SFC20240719-M202	0.30 ± 0.52
	Aspergillus sydowii	SFC20240719-M203	0.30 ± 0.52
	Geotrichum candidum	SFC20240719-M211	0.30 ± 0.52
	Clonostachys chloroleuca	SFC20240719-M212	0.30 ± 0.52
Mature	*Nigrograna* sp. 2	SFC20240812-M184	0.60 ± 0.00
	Nigrograna hydei	SFC20240812-M185	0.60 ± 0.00
	Nigrograna kunmingensis	SFC20240812-M186	0.60 ± 0.00
	Periconia sp. 1	SFC20240812-M187	0.60 ± 0.00
	Cladosporium marinisedimentum	SFC20240812-M190	0.60 ± 0.00
	Penicillium maximae	SFC20240812-M199	0.60 ± 0.00
	Neosetophoma sp.1	SFC20240812-M213	0.60 ± 0.00
	Neodidymella thailandica	SFC20240812-M191	0.30 ± 0.52
	Geotrichum candidum	SFC20240812-M192	0.30 ± 0.52
	Penicillium sumatraense	SFC20240812-M196	0.30 ± 0.52
	Trichoderma guizhouense	SFC20240812-M168	0.30 ± 0.52
	Trichoderma insigne	SFC20240812-M175	0.30 ± 0.52
	Fusarium acuminatum	SFC20240812-M156	0.30 ± 0.52
	Penicillium koreense	SFC20240812-M181	0.30 ± 0.52
	Paraconiothyrium cyclothyrioides	SFC20240812-M188	0.30 ± 0.52
	Curvularia sp. 1	SFC20240812-M189	0.30 ± 0.52
	Clonostachys linzhiensis	SFC20240812-M193	0.30 ± 0.52
	Penicillium sumatraense	SFC20240812-M195	0.30 ± 0.52
	Aspergillus fumigatus	SFC20240812-M197	0.30 ± 0.52
	Penicillium yuyongnianii	SFC20240812-M198	0.30 ± 0.52
	Aspergillus creber	SFC20240812-M206	0.30 ± 0.52
	Trichoderma sp. 7	SFC20240812-M219	0.30 ± 0.52
	Sporobolomyces carnicolor	SFC20240812-M145	0.15 ± 0.64
